# The Impact of SGLT2 Inhibitors on Pulmonary Artery Pressures and Pulmonary Hemodynamics in Patients With Heart Failure: A Systematic Review

**DOI:** 10.1155/cdr/6649731

**Published:** 2025-10-21

**Authors:** Kritick Bhandari, Maria Qadri, Rochak Dhakal, Sagun Ghimire, Gyanendra Jora, Santosh Basyal, Sanjit Kumar Shah

**Affiliations:** ^1^Department of Internal Medicine, KIST Medical College and Teaching Hospital, Lalitpur, Bagmati, Nepal; ^2^Department of Internal Medicine, Jinnah Sindh Medical University, Karachi, Sindh, Pakistan; ^3^Department of Applied Computing, Michigan Technological University, Houghton, Michigan, USA; ^4^Department of Internal Medicine, Nepal Medical College, Kathmandu, Bagmati, Nepal

**Keywords:** heart failure, heart failure with preserved ejection fraction, heart failure with reduced ejection fraction, pulmonary artery pressures, pulmonary hemodynamics, pulmonary hypertension, SGLT2 inhibitors

## Abstract

**Background:**

Heart failure is a major global health burden associated with high morbidity and mortality. Elevated pulmonary artery pressures (PAP) are linked to worse outcomes in heart failure patients. Sodium–glucose cotransporter 2 (SGLT2) inhibitors, initially developed for diabetes, have demonstrated cardiovascular benefits, but their specific effects on pulmonary hemodynamics remain unclear.

**Methods:**

This systematic review analyzed randomized controlled trials and observational cohort studies evaluating the effects of SGLT2 inhibitors on mean pulmonary artery pressure (mPAP) and pulmonary artery systolic pressure (PASP) in heart failure patients. A comprehensive search of PubMed, Embase, Cochrane Library, and Scopus databases was conducted until August 2024. Studies were appraised using PRISMA and AMSTAR guidelines, the Cochrane bias tool, and the Newcastle–Ottawa Scale.

**Hypothesis:**

SGLT2 inhibitors reduce PAPs in heart failure patients, leading to beneficial pulmonary hemodynamic effects.

**Results:**

Six studies (four RCTs and two observational; *n* = 346) were included. At rest, pooled analysis of three trials showed a significant reduction in mPAP (MD −1.41 mmHg; 95% CI −2.80 to −0.01; *p* = 0.05; *I*^2^ = 12%). During exercise, two studies demonstrated a nonsignificant reduction in mPAP (MD −3.12 mmHg; 95% CI −7.60 to 1.36; *p* = 0.17; *I*^2^ = 54%). For PASP, pooled analysis of four studies suggested a nonsignificant reduction (MD −6.72 mmHg; 95% CI −14.98 to 1.54; *p* = 0.11; *I*^2^ = 96%), but sensitivity analysis excluding one outlier yielded a significant effect (MD −2.76 mmHg; 95% CI −4.99 to −0.53; *p* = 0.02; *I*^2^ = 0%). Secondary outcomes included significant reductions in PCWP, PADP, and NT-proBNP.

**Conclusion:**

SGLT2 inhibitors demonstrate beneficial effects on pulmonary pressures and hemodynamics in patients with heart failure, with consistent trends toward lower mPAP, PASP, and PCWP. Although results are influenced by study heterogeneity, the overall evidence suggests meaningful hemodynamic improvements. Larger, long-term randomized trials are warranted to clarify subgroup effects (HFrEF vs. HFpEF and dapagliflozin vs. empagliflozin) and establish clinical implications.

## 1. Background

Heart failure (HF) remains one of the most significant health problems of the western world, affecting over 6.7 million Americans over the age of 20, and the prevalence is expected to rise to 11.4 million by 2050 [1]. Patients with HF are frequently classified based on ejection fraction (EF) into three main categories: heart failure with reduced ejection fraction (HFrEF) (EF < 40%), midrange EF (EF between 40% and 49%), and heart failure with preserved ejection fraction (HFpEF) (EF > 50%) [2]. Numerous pharmacological and mechanical interventions have been developed to combat HF, but these have mostly helped improve the quality of life and prognosis of patients with HFrEF [3]. Management lags by a significant amount for patients with HFpEF, despite both having similar prognoses. The pulmonary circulation has been gaining attention in recent years as a potential therapeutic target for HF patients. This is because elevated pulmonary artery pressures (PAPs) have been strongly associated with worse prognosis and increased hospitalization and mortality in HF patients [4]. Many patients with diastolic dysfunction have chronically elevated pulmonary pressures, which results in pulmonary vascular remodeling, pulmonary hypertension (PH), and right ventricular (RV) dysfunction. This increase in RV pressure and RV dysfunction ultimately results in worse prognosis in HF patients [5, 6]. Recent evidence suggests that even subtly elevated mean pulmonary artery pressure (mPAP) can pose a significant threat in HF populations, and as such, greater emphasis on early diagnosis of PH has been placed [7]. Despite being a major public health issue in HF populations, a proper consensus regarding the diagnosis and management of PH is still an area that remains quite unexplored [8]. One of the emerging drugs in HF therapy that has gained significant attention is sodium–glucose cotransporter 2 inhibitors (SGLT2 inhibitors). Originally designed as an antidiabetic medication, this group of drugs has shown promising outcomes in cardiovascular improvements, and its utility in HF patients is being explored [2]. Although SGLT2 inhibitors have demonstrated promising results in reducing cardiovascular events and improving HF outcomes, their specific effects on PAP remain underexplored. Given the limitations of existing recommendations regarding the effect of SGLT2 inhibitors in pulmonary hemodynamics, there was a growing need to explore the potential of these drugs in the pulmonary circulation. This could help address gaps in treatment, offering a more comprehensive strategy for managing HF, particularly in patients with elevated PAP.

This systematic review is aimed at critically exploring and assessing the effects of SGLT2 inhibitors on PAP in patients with HF. The goal is to evaluate the therapeutic efficacy of SGLT2 in the modulation of pulmonary pressures and pulmonary hemodynamics and to assess their impact on overall cardiac function, so as to analyze their potential capacity to improve outcomes in HF patients.

## 2. Methods

### 2.1. Study Protocol

This systematic review was conducted in accordance with the Preferred Reporting Items for Systematic Review and Meta-Analysis (PRISMA) statement and AMSTAR (assessing the methodological quality of systematic reviews) guidelines [9, 10]. The PRISMA and AMSTAR checklist is presented in supporting information (Appendices S1 and S4). Additionally, the review methods were established prior to the conduct of the review, and we have registered the systematic review in the database of PROSPERO with ID: CRD42024583533.

### 2.2. Study Selection

Publications examining mPAP and pulmonary artery systolic pressure (PASP) change values during both rest and physical exercise in HF patients taking SGLT2 inhibitors were identified by a Medline search using a comprehensive search strategy (Appendix S2). Systematic searches of online databases such as Embase, PubMed, Scopus, and Cochrane Library until 22 August 2024 were conducted to find the relevant articles. The search strategy consisted of Medical Subject Headings (MeSH) terms, keywords, and search terms such as: “Heart failure”, “Sodium-Glucose Transporter 2 Inhibitors”, “Pulmonary wedge pressure”, “Heart failure, Diastolic”, and “Heart failure, Systolic”. Appropriate Boolean operators “AND”/”OR” were used between the aforementioned terminologies. We used Zotero to store the studies that were considered eligible, with limitations only to publications in the English language, which could introduce language bias. The details of the search strategy are shown in the supporting information (Appendix S2).

Original randomized control trials (RCTs), observational cohort studies, and case-controlled studies on the impact of SGLT2 inhibitors versus placebo or optimum medical therapies (OMTs) on mPAP or PASP changes were included. RCTs were prioritized for their ability to establish causality and minimize bias through randomization. Observational studies, including cohort and case-control designs, were included to complement RCT findings by reflecting real-world clinical practices and providing insights into broader patient populations and longer follow-up periods. A secondary search reviewed the reference lists of relevant papers. When studies could not be retrieved, their authors were contacted to request a copy of the publication. Duplicate screening was done by Zotero. Two reviewers (K.B. and S.G.) independently screened the studies identified by the searches after the duplicate removal. Differences over the inclusion of studies were resolved by consensus reached after discussion with a third reviewer. The studies were assessed based on key characteristics such as intervention (SGLT2 inhibitors), population (HF patients), and outcomes (PAPs). The final selection was made by comparing these characteristics against predefined inclusion and exclusion criteria.

### 2.3. Search Techniques

Using the population, intervention, comparison, and outcome (PICO) criteria, a thorough review of the literature was carried out. The details of the search technique are given in the supporting information (Appendix S2).

Inclusion and exclusion criteria:

To accomplish our research objectives, we defined precise inclusion and exclusion criteria.  Table 1 provides an overview of our criteria.

### 2.4. Quality Appraisal

We made use of a variety of quality assessment tools to ensure the validity of the papers we chose. For randomized clinical trials, we used the Cochrane risk of bias tool assessment. The Newcastle–Ottawa tool scale was used to evaluate observational/case-control studies. Only studies with low and intermediate risk of bias were included in the review. The details of quality assessment are presented in the supporting information (Appendix S3).

### 2.5. Data Extraction and Management

Studies obtained from the electronic databases, supplementary sources, and manual searching were exported to Zotero in a compatible format. Duplicate articles were screened first by Zotero and then manually. Duplicates were then recorded and removed. For multiple publications of the same data in more than one journal, the most inclusive, comprehensive studies, with larger sample sizes and the most recent ones were considered. Basic data extraction was conducted in a Microsoft Excel spreadsheet. Two reviewers (K.B. and M.Q.) extracted the data independently, and a third reviewer conducted a final revision of the collected data.

The spreadsheet captured key information, including study design, sample size, follow-up duration, publication date, participant demographics (age, sex, and ethnicity), drug used, HF type, baseline left ventricular EF, baseline NT-proBNP levels, baseline New York Heart Association (NYHA) functional class, comorbidity at baseline, and outcomes (main and additional).

#### 2.5.1. Data Synthesis and Analysis

The effect measure used for the main outcome (change in mPAP or PASP) and all additional outcomes (pulmonary artery diastolic pressure [PADP], pulmonary vascular resistance [PVR], pulmonary capillary wedge pressure [PCWP], and NT-proBNP) is the mean difference.

Due to the presence of missing data across some studies required for meta-analysis, we opted to tabulate findings from all the studies and create forest plots only from those studies that provided complete datasets. Where possible, we attempted to fill these gaps by extracting missing data following guidelines outlined in the Cochrane Handbook for Systematic Reviews Version 6.5 [11]. For studies that reported changes only in PASP, we derived mPAP using the internationally recognized formula: mPAP = 0.61 × PASP + 1.95 mmHg. The SD for mPAP can also be derived from the formula for PASP. Since the mPAP formula involves a linear transformation of PASP, the standard deviation of mPAP can be obtained as SD_mPAP_ = 0.61 × SD_PASP_. This approach allowed us to synthesize the available data meaningfully while acknowledging the limitations posed by the incomplete datasets. However, the precision of these estimated effects could not be fully reliable, and this lack of accurate data may have affected the robustness of our synthesis. Thus, we did not use the derived data while generating forest plots and only used them in tabular form.

We used tables to organize and visually display the results of individual studies. These tables summarized key characteristics of the included studies, such as study population, demographics, baseline values, intervention type (e.g., empagliflozin [EMPA] vs. dapagliflozin [DAPA]), and main outcomes (e.g., changes in mPAP and PASP) and secondary outcomes (PADP, PCWP, PVR, and NT-proBNP). To explore potential causes of heterogeneity among study results, we conducted subgroup analyses. Specifically, we compared rest and exercise hemodynamics to determine whether the effects of SGLT2 inhibitors differed based on the physiological state during measurements. Additionally, we performed subgroup analyses comparing the effects of EMPA and DAPA to assess any variations in their impact on PAPs. These analyses helped identify whether differences in treatment or hemodynamic conditions contributed to heterogeneity in the study outcomes.

To assess the risk of bias due to missing results in the synthesis, we considered potential reporting biases, such as selective outcome reporting or incomplete reporting of key data. We reviewed each study to determine if all prespecified outcomes were fully reported, as per their protocols or methods sections. Additionally, we checked for discrepancies between reported outcomes in the main text and supporting information. Studies that failed to report key outcomes, such as changes in PAPs (mPAP or PASP), were flagged as potentially having a risk of bias.

In cases where data appeared to be missing or selectively reported, we attempted to obtain missing data by consulting supporting information or by referencing the Cochrane Handbook for Systematic Reviews to fill in gaps, particularly regarding methodological details. However, if missing data could not be retrieved, these studies were noted, and their potential impact on the overall conclusions was considered in the narrative synthesis.

### 2.6. Outcomes

#### 2.6.1. Primary Outcome

1. Change in mPAP: mPAP reflects the average pressure within the pulmonary artery. It is measured in millimeters of mercury and is calculated using the formula: mPAP = 1/3 PASP + 2/3 PADP. It can also be estimated from PASP using the formula: mPAP = 0.61 PASP + 1.95 mmHg.

Data on mPAP from all available studies were sought, and mPAP values on baseline and the final follow-up were recorded, regardless of the method of measurement (e.g., direct hemodynamic measurements via right heart catheterization (RHC) or noninvasive echocardiographic estimation).

2. Change in PASP: The peak pressure in the pulmonary artery during the systolic phase of the cardiac cycle.

Data on PASP from all available studies were sought, and PASP values on baseline and the final follow-up were recorded, regardless of the method of measurement (e.g., direct hemodynamic measurements via RHC or noninvasive echocardiographic estimation).

#### 2.6.2. Secondary Outcomes

1. Change in PADP: An additional measure of PAP, collected when available. In clinical studies of HF, mPAP and PASP tend to be reported more frequently than PADP. To avoid potential gaps or inconsistencies in the studies, PADP was considered a secondary outcome in our study. Further, mPAP is often derived using PASP and PADP, so including all three parameters could introduce redundancy.
2. Change in PVR: Represents the pressure in the arteries that supply blood to the lungs.
3. Change in PCWP: An indirect measure of the left atrial pressure, relevant in assessing HF status.
4. Change in NT-proBNP levels: NT-proBNP is a biomarker often used to assess the severity of HF and its progression.

## 3. Results

### 3.1. Study Selection

**Figure 1:**
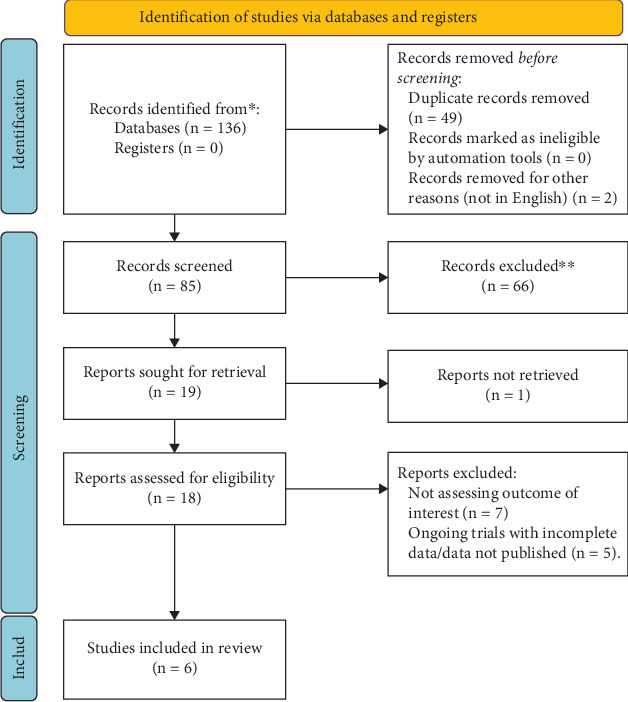
PRISMA (Preferred Reporting Items for Systematic Reviews and Meta-Analysis) diagram showing study selection process.

Altogether, 136 articles were obtained from the databases of Embase, PubMed, Scopus, and Cochrane Library from inception until 22 August 2024. From the initial search results, 49 duplicate articles were removed and two articles were removed because they were not in the English language. From the remaining 85 articles, 66 articles were removed by screening the title and abstract. Full-text review was done thoroughly on the remaining 19 articles, out of which 13 articles were excluded based on the eligibility criteria. Finally, six full-text articles were included in the analysis. The PRISMA diagram tailoring the details of the study selection process is shown in Figure 1.

### 3.2. Study Characteristics

These six studies [12–17] mentioned below (Table 2) included 346 HF patients in total, with their sample size ranging from 38 to 78. The studies include RCTs and cohort studies conducted from the year 2020–2024. Most of the studies have reported a mean age of the patients to lie between the mid-60s and late 70s. All studies, with the exception of Reddy et al. [13], comprised a predominant male population. However, in the study by Jariwala and Gururaj [16], the demographic data could not be extracted. Only three of the six retrieved studies had any available data on ethnicity [13–15] and those were primarily white populations. Hypertension, atrial fibrillation, and diabetes mellitus were the main comorbidities in the population. In addition to that, obesity, ischemic heart diseases, chronic kidney disease, chronic obstructive pulmonary disease, and anemia were reported. All included participants were on OMT for HF, while only those participants included in the study by Omar et al. [14] and Correale et al. [17] had additional glucose lowering therapies as baseline medications. Of the six, three studies measured PAPs using echocardiography [12, 16, 17], whereas in two studies, RHC was performed [13, 14] and one study used an implanted PAP sensor (CardioMEMS) (Table 3) [15]. Only one of our studies involved HFpEF [13] patients and two studies involved a population with HFrEF [12, 14], while the rest included HF irrelevant of EF. The most prevalent NYHA classes among the subjects in order of frequency were II, III, and IV. In addition, levels of NT-proBNP varied from 100 to 200 pg/mL to values as high as 2300 pg/mL. Two studies [12, 13] used DAPA as the drug in the intervention group. EMPA was the intervention group drug in three studies [14–16]. Correale et al. [17] used an SGLT2 inhibitor in their intervention group, but this was not specified. The six studies included varied significantly in follow-up duration, assessment techniques, and participant backgrounds, introducing potential heterogeneity in the study.

### 3.3. Risk of Bias Within Studies

The risk of bias for each included study was assessed using both the Cochrane Risk of Bias 2 (ROB 2) tool for randomized controlled trials and the Newcastle–Ottawa Scale (NOS) for nonrandomized studies. Detailed assessments, including domain-specific evaluations of selection, comparability, and outcome/exposure risks, are provided in the supporting information (Appendix S3). The ROB 2 tool evaluates bias across several domains including the randomization process, deviations from intended interventions, and outcome measurement, while the NOS assesses the quality of cohort and case-control studies based on selection, comparability, and exposure/outcome ascertainment. All of the studies had a low or intermediate risk of bias and thus were included in our review.

### 3.4. Result

#### 3.4.1. Primary Outcome

**Figure 2:**
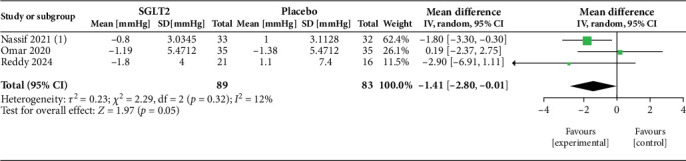
Effect of SGLT2 inhibitors versus placebo/standard care on mean pulmonary artery pressure (mPAP) at rest.

**Figure 3:**
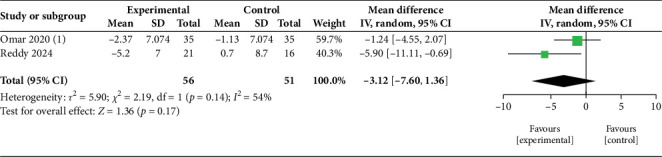
Effect of SGLT2 inhibitors versus placebo/standard care on mean pulmonary artery pressure (mPAP) during exercise.

**Figure 4:**
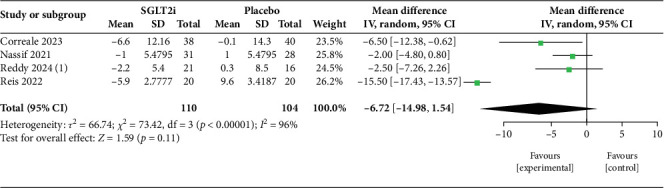
Effect of SGLT2 inhibitors versus placebo on pulmonary artery systolic pressure (PASP).

**Figure 5:**
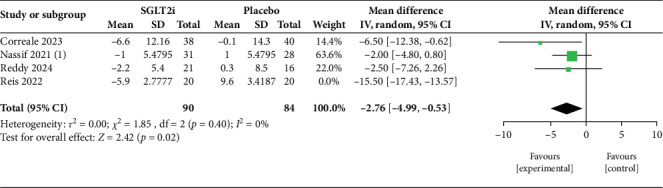
Leave-one-out sensitivity analysis for pulmonary artery systolic pressure (PASP).

Our study included several papers that have explored the efficacy of two SGLT2i drugs separately: DAPA and EMPA. The primary outcomes of our review have been tabulated in Table 4 and demonstrated in Figures 2, 3, 4, and 5. These trials compared the intervention group with either placebo or optimum treatment, concerning their effects on cardiac hemodynamics. The principal findings suggest that SGLT2 inhibitors, compared with placebo or standard care, are associated with meaningful reductions in pulmonary pressures, although the strength and consistency of these effects vary according to clinical setting (rest vs. exercise), study design, and drug type.

##### 3.4.1.1. mPAP

Three studies [13–15] contribute to the pooled estimate of changes in mPAP at rest (Figure 2). The random-effects meta-analysis shows a reduction in mPAP with SGLT2i (MD = −1.41 mmHg; 95% CI: −2.80 to −0.01; *p* = 0.05). Between-study heterogeneity is low (*I*^2^ = 12%; *τ*^2^ = 0.23; *χ*^2^ = 2.29, df = 2; *p* = 0.32), supporting a fairly consistent direction of effect across studies. Two studies [13, 14] are pooled to show the effect of exercise on mPAP hemodynamics (Figure 3). The summary effect is not statistically significant (MD = −3.12 mmHg; 95% CI: −7.60 to 1.36; *p* = 0.17). Heterogeneity is moderate (*I*^2^ = 54%; *τ*^2^ = 6.90; *χ*^2^ = 2.19, df = 1; *p* = 0.14), suggesting some variability in exercise hemodynamic response, driven largely by the study showing a larger reduction, yet still insufficient for a confident pooled difference. Statistical heterogeneity also likely reflects differences in population characteristics (HFrEF vs. HFpEF).

##### 3.4.1.2. PASP

For PASP, four studies [12, 13, 15, 17] were included in the pooled analysis (Figure 4). The combined effect suggested a reduction with SGLT2i but did not achieve statistical significance (MD = −6.72 mmHg; 95% CI: −14.98 to 1.54; *p* = 0.11). Heterogeneity was very high (*I*^2^ = 96%), reflecting wide methodological and population differences between trials, particularly between DAPA- and EMPA-based studies. Importantly, the leave-one-out sensitivity analysis (Figure 5) demonstrated that exclusion of a single high-influence study markedly reduced heterogeneity (*I*^2^ = 0%) and yielded a statistically significant reduction in PASP (MD = −2.76 mmHg; 95% CI: −4.99 to −0.53; *p* = 0.02). This highlights that while overall results are sensitive to study-level variability, the underlying direction of effect consistently favors SGLT2i therapy.

Taken together, these findings support the conclusion that SGLT2 inhibitors lower pulmonary pressures in patients with HF, though the magnitude and statistical robustness of the effect vary. The consistency of effect direction, the low heterogeneity in resting mPAP, and the robustness of the PASP signal after sensitivity analysis lend confidence that SGLT2 inhibitors contribute meaningfully to hemodynamic improvement in HF. Nevertheless, further high-quality, head-to-head randomized trials are required to directly compare DAPA and EMPA, standardize exercise protocols, and extend follow-up to determine long-term clinical implications of pulmonary pressure reduction.

##### 3.4.1.3. Subgroup Analysis

A. Hemodynamics at rest and exercise:

The analysis of pulmonary hemodynamic parameters in patients with HF during rest and exercise revealed a trend toward greater reduction in mPAP and PASP (Table 5). At rest, pooled analysis of three studies [13–15] (Figure 2) demonstrated a modest but borderline significant reduction in mPAP with SGLT2i (MD = −1.41 mmHg; 95% CI: −2.80 to −0.01; *p* = 0.05). Heterogeneity was low (*I*^2^ = 12%), suggesting reasonable consistency across trials despite differences in patient populations and measurement techniques. Exercise hemodynamics showed nonsignificant reductions in mPAP. In the study by Reddy et al. [13], the patients underwent exercise to volitional exhaustion, and values were measured via echocardiography; DAPA caused a substantial decrease in mPAP compared to placebo during exercise (−5.9 mmHg, *p* = 0.02), with a comparable reduction in PASP (−5.3 mmHg); however, the latter did not reach statistical significance. EMPA, in Omar et al.'s [14] study, showed a marginally significant reduction in mPAP during exercise (−2.37 mmHg, *p* = 0.056), suggesting a trend toward improvement. They used a cycle ergometer exercise test until volition and calculated pulmonary pressures via RHC. The pooled effect across two studies [13, 14] (Figure 3) showed a nonsignificant reduction in mPAP (MD = −3.12 mmHg; 95% CI: −7.60 to 1.36; *p* = 0.17), with moderate heterogeneity (*I*^2^ = 54%). This reflects variability in exercise protocols and hemodynamic responses, although both studies favored SGLT2i over placebo. The trend toward larger benefit under stress conditions aligns with the hypothesis that these agents may improve pulmonary vascular compliance or left heart unloading when circulatory demand is increased. More studies are necessary to find out the exact impact of SGLT2 inhibitors on pulmonary hemodynamics during exercise. Further, such changes in pulmonary hemodynamics during exercise may be normal for HF pathophysiology and not specifically due to SGLT2 inhibitors. The heterogeneity due to the different types of exercise tests used and the method of measuring the variables should also be considered.

B. DAPA Versus EMPA:

Two notable studies with DAPA as the intervention drug, Reis et al. [12] and Reddy et al. [13], demonstrated statistically significant impacts on the primary outcomes. Reis et al. [12] found a 5.9 mmHg reduction in PASP in the intervention group compared to a 9.6 mmHg increase in PASP in the placebo group on a 6-month follow-up. The calculated/derived mPAP in their study reduced from 24.27 to 20.33 mmHg (mean reduction of 3.94 mmHg) in the intervention group compared to a 5.86 mmHg increase in the control group (20.13 mmHg at baseline vs. 25.99 mmHg at follow-up). Reddy et al. [13] demonstrated that DAPA had a significant impact on mPAP during exercise with a mean of 5.9 mmHg reduction in mPAP after 24 weeks compared to placebo. Even during rest, mPAP value was 2.8 mmHg lower in the intervention compared to the control (1.8 mmHg reduction in mPAP in the intervention group compared to a 1.0 mmHg increase in mPAP in the control) after 24 weeks follow-up; however, the resting hemodynamics were not statistically significant. Similarly, the PASP at rest was 2.6 mmHg lower in the DAPA group compared to placebo after 24 weeks (*p* = 0.27). But, the exercise PASP had a statistically insignificant (*p* = 0.08) reduction, 5.3 mmHg lower in the DAPA group compared to the placebo.

EMPA has shown more variable results. In the study by Nassif et al. [15], EMPA was associated with a 1.9 mmHg reduction in mPAP (intervention vs. control group); however, this finding was not statistically significant. EMPA also reduced PASP compared to placebo, but the reduction was not statistically significant. Omar et al. [14] reported modest reductions in mPAP in the intervention group during exercise, but this was a marginally significant finding. However, the resting hemodynamics in their study showed conflicting findings, with greater reduction of mPAP in the placebo group (−1.38 mmHg) compared to intervention (−1.19 mmHg), but this was not statistically significant. Jariwala and Gururaj [16] found that the PASP remained rather constant while comparing the efficacy of adding EMPA on HF patients under OMT with those on OMT alone. The effect of EMPA, as seen in the studies by Omar et al. [14] and Nassif et al. [15], produced modest effects. This could be because these trials were conducted for a shorter duration (12 weeks) compared to the trials by Reis et al. and Reddy et al. [12, 13], which took around 6 months. In contrast, Jariwala and Gururaj [16] reported no change in PAPs even after 9 months of therapy.

Most trials demonstrate a drop in PAPs when SGLT2 therapies are used compared to controls; however, there are still inconsistencies in their effectiveness, as demonstrated by the variability in responses according to the duration of therapy (Table 6). Nevertheless, the consistent reduction of pulmonary pressures with this group of drugs over most of the trials could not be solely by chance, and further research is required to build upon these findings. However, since the studies varied in terms of baseline characteristics and follow-up duration, the findings should be carefully speculated upon, and further research is warranted before we can formulate a solid conclusion regarding the superiority of a particular SGLT2i group. This variability suggests potential differences in how these drugs affect pulmonary pressures, emphasizing the need for more targeted research.

C. HFrEF Versus HFpEF:

Because of the scarcity of research studies directly comparing HFrEF and HFpEF patients, we could not extract the comparative efficacy of SGLT2 inhibitors between these subgroups. From limited data derived from two studies, it is seen that DAPA could reduce pulmonary pressure in both HF populations (Table 7). In HFrEF, Reis et al. [12] reported a significant PASP reduction with DAPA (−5.9 mmHg, *p* < 0.001), whereas the control group showed an increase (+9.6 mmHg). Similarly, in the HFpEF subgroup, Reddy et al. [13] found a significant reduction in mPAP during exercise (−5.9 mmHg, *p* = 0.02) and a trend toward PASP reduction (−5.3 mmHg, *p* = 0.08), though resting hemodynamics were not statistically significant. In HFpEF, EMPA showed minimal effects, with Omar et al. reporting nonsignificant PASP reductions at rest (−1.19 mmHg, *p* = 0.23) and during exercise (−2.37 mmHg, *p* = 0.056). The control group experienced comparable changes, indicating no substantial benefit. There was no data for the efficacy of EMPA in the HFrEF group. Despite the scarcity of data, it is seen that the pulmonary hemodynamics have a consistent trend toward reduction with SGLT2 inhibitors intervention compared to control.

#### 3.4.2. Secondary Outcome

SGLT2 inhibitor drugs, such as DAPA and EMPA, have shown significant effects on various hemodynamic parameters when compared to placebo in patients with HF (Table 8).

DAPA demonstrated notable improvements in PCWP and NT-proBNP levels. In the study by Reddy et al. [13], DAPA reduced PCWP significantly, with a change of −3.5 mmHg at rest and −6.1 mmHg during exercise, compared to minimal changes in the placebo group. These reductions were statistically significant (*p* = 0.03 and *p* = 0.02, respectively). NT-proBNP levels also showed a greater reduction with DAPA, highlighting its beneficial impact on HF biomarkers. Only Reddy et al. [13] demonstrated the impact over PVR. The changes in PVR were not significant, with only minimal variations between the DAPA and placebo groups.

EMPA also demonstrated significant improvements in key outcomes. In the study by Omar et al. [14], EMPA reduced PADP during both rest and exercise. At rest, the change was minimal (from 14 to 13 mmHg), but during exercise, the reduction was more pronounced, dropping from 32 to 29 mmHg. PCWP also showed significant reductions with EMPA, with a decrease of −2.16 mmHg at rest and −4.14 mmHg during exercise, compared to smaller changes in the placebo group. Additionally, NT-proBNP levels were significantly reduced, as seen in Nassif et al. [15], where a greater proportion of patients on EMPA experienced a ≥ 20% reduction in NT-proBNP levels (34% in the EMPA group vs. 7% in the placebo group, *p* = 0.01).

Overall, both DAPA and EMPA demonstrated improvements in reducing PCWP and NT-proBNP levels, with DAPA showing a tendency toward stronger effects on PCWP during exercise and EMPA showing significant reductions in NT-proBNP levels, indicating their potential benefits in managing pulmonary pressures and HF symptoms. Reis et al. [12] demonstrated that DAPA significantly enhanced RV–PA coupling over 24 weeks, as evidenced by an increase in TAPSE/PASP from 0.52 to 0.66. In contrast, the control group showed deterioration in coupling. Similarly, Jariwala and Gururaj [16] found that EMPA combined with OMT led to a notable increase in TAPSE/PASP (0.252→0.300), with the change largely attributed to improved RV function rather than reduced PASP. Correale et al. [17] corroborated these findings, reporting a striking rise in TAPSE/PASP (0.63→0.88) with SGLT2 inhibitor therapy, driven by both increased TAPSE and decreased PASP. Likewise, in the study by Reddy et al. [13], while no significant benefit in RV–PA coupling was observed at rest, exercise testing revealed a statistically significant improvement in RVs'/PA Ea with DAPA (*p* = 0.04), suggesting enhanced physiological adaptability under stress.

## 4. Discussion

Our systematic review has evaluated the efficacy of SGLT2 inhibitors in improving PAPs and pulmonary hemodynamics in the HF population. The data comes from four RCTs and two observational studies comparing SGLT2 inhibitors with the control group. The principal findings of our analysis indicate that SGLT2 inhibitors, when compared to placebo, demonstrate a meaningful impact on reducing PAPs and improving pulmonary hemodynamics in the HF population. It is imperative to conduct more structured research to derive an exact conclusion on the efficacy of DAPA versus EMPA and their impact on rest versus exercise and HFpEF versus HFrEF. This systematic review represents the most comprehensive examination to date, comprising a cohort of 346 participants. It encompasses a comprehensive review of all existing data regarding the change in PAPs and pulmonary hemodynamics in the context of SGLT2 inhibitors therapy.

Numerous studies have consistently highlighted the positive effects of SGLT2 inhibitors on pulmonary pressures, reinforcing the findings from the studies included in our review (Table 9). A statistically significant decrease in key measures like PASP, mPAP, PADP, and NT-proBNP levels was observed across all the observational studies [18–24], often within relatively short follow-up periods. These reductions are comparable to the outcomes seen in controlled trials, which similarly demonstrated improvements in pulmonary hemodynamics. What stands out from these observational studies is the consistency in the direction of the results, despite having some differences in patient populations, follow-up duration, and study designs. Likewise, a randomized controlled trial exploring the efficacy of combining sacubitril/valsartan with DAPA in patients with pulmonary hypertension secondary to left heart disease (PH-LHD) found that the group that received both drugs showed notable improvements in mPAP and PASP in comparison to the control group that took only sacubitril/valsartan [25]. Such consistency in reducing PASP and mPAP, along with improvements in HF biomarkers, as seen in several RCTs and observational studies, suggests that SGLT2 inhibitors offer robust and replicable benefits across diverse real-world settings. This reinforces the evidence supporting the broader use of these agents for improving pulmonary hemodynamics in HF patients.

Pulmonary hemodynamic parameters are crucial for evaluating and treating patients with HF [26–29]. They offer essential information regarding the hemodynamic conditions of the pulmonary circulation, which frequently suffers in HF due to increased left-sided filling pressures, resulting in PH [30]. The pathophysiological distinctions between combined precapillary postcapillary pulmonary hypertension (CpcPH) and isolated postcapillary pulmonary hypertension (IpcPH) are also clarified by such hemodynamic measures. We looked at the significance of a number of pulmonary hemodynamic indicators, including mPAP, PASP, PADP, PCWP, PVR, and NT-proBNP, highlighting their influence on HF patients and their prognostic value. One important indicator of cardiac stress and volume overload is NT-proBNP, whose elevated levels are associated with worse outcomes in HF via raising pulmonary pressures and impairing functional status [31, 32]. mPAP > 20 mmHg defines PH and correlates with poor prognosis due to elevated pulmonary pressures and RV dysfunction [7, 30, 33, 34]. PASP and PADP are also valuable: elevated PASP reflects impaired functional capacity and higher mortality risk, while increased PADP indicates rising left atrial pressure from LV dysfunction [31, 35]. PCWP serves as a surrogate for left atrial pressure; its elevation signals fluid overload, pulmonary congestion, and worse HF outcomes [35, 36]. PVR reflects pulmonary vascular disease, impaired RV function, and is strongly predictive of adverse prognosis. Specifically, PVR levels greater than 3 Wood units are commonly found in patients with significant pulmonary vascular disease [7, 37], affecting treatment decisions and predicted outcomes. Certain hemodynamic measures are closely linked to clinical outcomes, including mortality and major adverse cardiac events (MACE). For example, variations in mPAP are closely tied to negative clinical outcomes in patients with pulmonary arterial hypertension (PAH), showcasing considerable prognostic capability [26]. Likewise, the diastolic pressure gradient (DPG) serves as a significant predictor of mortality for patients with PH stemming from left heart disease [29]. A reduction in PCWP is significant for managing PH and HF. Lower PCWP can improve cardiac filling and reduce pulmonary congestion, potentially enhancing exercise capacity and reducing hospitalization rates [38]. We found that SGLT2i shows a generally favorable trend toward improved RV–PA coupling metrics (Table 8) [12, 13, 16, 17]. Reddy et al. [13], hypothesized that a decrease in exercise PCWP with SGLT2 inhibitors is probably a major mediator of improved pulsatile pulmonary vascular load and RV–PA coupling during exertion. A key limitation in the interpretation of RV–PA coupling is the heterogeneity in how the parameter was assessed. A key limitation is heterogeneity in assessment: only Reddy et al. [13] used RVs'/Ea, a load-independent metric, whereas others [12, 16, 17] relied on TAPSE/PASP, a less reliable surrogate under varying RV geometry or load. Standardized use of RVs'/Ea in future trials may yield clearer insights into the cardiopulmonary benefits of SGLT2 inhibitors.

Implementing invasive hemodynamic evaluations can improve risk prediction models, facilitating customized management approaches for HF patients [29]. PAP-guided HF therapy has demonstrated favorable outcomes in reducing HF-related hospitalization [39]. Notably, two trials, the CHAMPION [40] and GUIDE-HF [41], reported significant reductions in HF-related hospitalization. Another prospective study analyzing PAP-guided therapy in patients with symptomatic HF after implant CardioMEMS showed that HF-related hospitalization decreased by 62% post- versus preimplant [42, 43]. A recent meta-analysis aggregated results from several of these randomized trials and concluded that using PAP monitoring to guide the treatment of HF patients reduces episodes of worsening HF and subsequent hospitalizations [44]. Ongoing monitoring of hemodynamic shifts also helps in treatment modifications, which can enhance patient outcomes [26, 45]. By incorporating these hemodynamic indicators into clinical routines, healthcare professionals can refine treatment strategies, boost survival rates, and elevate the overall quality of patient care.

The impact of SGLT2 inhibitors on Ipc-LHD and CPC-LHD remains an intriguing yet largely unexplored area of research. In IpcPH, the main cause of increased PAPs is the reverse transmission of raised left ventricular filling pressures. By promoting natriuresis and osmotic diuresis without neurohormonal activation, SGLT2 inhibitors reduce LV preload and pulmonary venous congestion [22, 46]. Several trials [13–15] support the unloading effect of SGLT2 inhibitors, with consistent reductions in PCWP and PADP (Table 8) observed within weeks of therapy, both at rest and during exercise. These consistent improvements in postcapillary pressures strongly support the utility of SGLT2 inhibitors in IpcPH. In contrast, CpcPH involves not only elevated left-sided pressures but also increased PVR (PVR ≥ 3 WU) due to vascular remodeling, endothelial dysfunction, and inflammation. The studies included in this review have failed to address the changes in PVR. Besides offering similar hemodynamic relief in CpcPH as in IpcPH, their additional impact on the pulmonary vasculature as shown in preclinical models implies possible advantages of SGLT2 inhibitors for both PH subgroups [47, 48]. Evidence regarding CpcPH is developing, but it is not yet very strong, reflecting the difficulties in reversing preexisting vascular issues. In one study of individuals with exercise-induced PH (a precursor to CpcPH), SGLT2 inhibitors notably reduced the slope of PCWP to cardiac output (PCWP/CO) during physical activity (3.9 ± 1.2 vs. 2.4 ± 1.2 mmHg/L/min) and improved the distance covered in a 6-min walk, indicating improved pulmonary vascular compliance [49]. The possibility of vascular remodeling reversal, suggested by animal models, warrants further investigation in dedicated CpcPH human trials. Because of the lack of adequate data related to CpcPH, the exact effects of these novel drugs on CpcPH could not be estimated, and this limitation would be an interesting target for future studies to bridge the gap of evidence.

SGLT2 inhibitors have shown significant benefits in cardiovascular outcomes, including reductions in HF-related hospitalizations, mortality, and symptom burden, in notable trials. The therapeutic effect of SGLT2 inhibitors in HF was first demonstrated in the empagliflozin cardiovascular outcome event trial in Type 2 diabetes mellitus patients removing excess glucose (EMPA-REG OUTCOME) trial [50] where it was noted that there was a significant reduction in the secondary endpoint of HF hospitalizations when compared with placebo. The canagliflozin and cardiovascular and renal events in Type 2 diabetes (CANVAS) [51], the dapagliflozin and cardiovascular outcomes in Type 2 diabetes (DECLARE TIMI) [52], and a pooled analysis by McGuire et al. [53] trials built upon this finding and came to similar conclusions in their study. These findings piqued the interest regarding the capacity of SGLT2 inhibitor to work beyond diabetes, tempting researchers to conduct trials directed toward HF outcomes. As such, several placebo-controlled clinical trials were then conducted to evaluate the effects of SGLT2 inhibitors in the chronic HF population with and without Type 2 diabetes. DAPA-HF (dapagliflozin and prevention of adverse outcomes in heart failure) [54] and EMPEROR-Reduced (empagliflozin outcome trial in patients with chronic heart failure with reduced ejection fraction) [55] studied patients with chronic HFrEF, and EMPEROR-Preserved (empagliflozin outcome trial in patients with chronic heart failure with preserved ejection fraction) [56] evaluated patients with chronic HFpEF. SGLT2 inhibitors were able to demonstrate a ~25% relative reduction in the composite end point of hospitalization for HF or cardiovascular death when compared with placebo in all these trials. The efficacy of SGLT2 inhibitors in exercise hemodynamics has been studied using cardiopulmonary exercise tests (CPETs), but the results varied across studies, with one study indicating improvements [57], while others showing no effects [58–60]. EMPA was associated with lower rates of hospitalization at 1 year compared with DAPA in a recent cohort study that compared the outcomes in reducing all-cause mortality and hospitalizations in patients with HF [61]. Another retrospective study by Hao et al. [62] also found that EMPA (vs. DAPA) resulted in greater increases in left ventricular EF and NYHA class improvements. But similar improvements in cardiovascular outcomes were observed between EMPA and DAPA compared with placebo in a recent meta-analysis [63]. These variations in findings could be due to a difference in adherence across studies. Thus, future researchers should also consider the adherence in the study population while conducting comparative research of the SGLT2 inhibitor drugs.

SGLT2 inhibitors are believed to reduce pulmonary pressures through several mechanisms. Inflammation is a notable contributor to HF. The anti-inflammatory potential of this novel drug stems from its ability to repress the endothelial expression of adhesion receptors, proinflammatory cytokines, and chemokines [64]. Specifically, DAPA can inhibit hyperglycemia-induced upregulation of intercellular adhesion molecule-1 (ICAM-1), while EMPA inhibits certain chemokines such as monocyte chemoattractant protein-1 (MCP-1). SGLT2 inhibitors have demonstrated a reduction in extracellular matrix production, suppression of collagen synthesis and remodeling, and inhibiting the proliferation and migration of vascular smooth muscle cells, highlighting their potential role in inhibiting pulmonary vascular remodeling [64–66]. SGLT2 inhibitors ensure the bioavailability of nitric oxide, a potent vasodilator, whose critical reduction leads to increased PAP seen in PH [67]. Their diuretic effect likely decreases pulmonary congestion, reducing the load on the right ventricle [20, 23]. The resultant volume contraction and hemoconcentration were attributed as the cause of reduction in cardiovascular mortality in a mediation analysis of the EMPA-REG outcome trial [68]. Besides their effect on the pulmonary circulation, numerous theories have tried to elucidate the cardioprotective mechanisms of SGLT2 inhibitors. These theories have explored the role of SGLT2 inhibitors in blood pressure lowering, cardiac energy metabolism, anti-inflammation, weight loss, diuresis/natriuresis, preventing cardiac remodeling, preventing ischemia/reperfusion injuries, decreasing oxidative stress, and so on. Although further research is necessary to explore the full potential of this novel drug, such multifaceted mechanisms offer a promising outlook for SGLT2 inhibitors in the management of HF and PH.

Multiple animal studies have provided more details on the cardioprotective mechanisms of SGLT2 inhibitors with respect to pulmonary hemodynamics. Both a general SGLT inhibitor and a specific SGLT2 inhibitor, canagliflozin, demonstrated targeted dilatation of pulmonary arteries in an in vitro analysis conducted on pulmonary and coronary arteries of diabetic mice [69]. Chowdhury et al. [70] showed that EMPA significantly reduced mPAP and RVSP in their studies on monocrotaline-induced PAH rat models [70]. In addition, Chowdhury et al. showed that EMPA decreased arterial wall thickening and pulmonary arteriolar muscularization in these rats, suggesting that SGLT2 inhibitors might prevent certain injurious structural pulmonary vascular changes. However, a similar study utilizing DAPA failed to demonstrate significant improvements in survival or vascular remodeling in monocrotaline-induced PAH rat models [71]. Yusuke et al. found pulmonary vascular remodeling in PH-LHD models was improved with tofogliflozin, suggesting SGLT2 inhibitors might be effective in treating PH-LHD [47]. While these studies provide diverse views on the efficacy of SGLT2 inhibitors in the pulmonary hemodynamics of HF patients, it must be noted that cross-pollination of research findings from animal studies to clinical practice in humans might not be an evidence-based approach in forming scientifically sound conclusions.

### 4.1. Strengths and Limitations

The results from this structured systematic review should be interpreted in the context of several potential limitations. One of the major limitations of this study is the small sample size and short follow-up duration, which restricts the generalizability of the findings. The study includes only a small number of heterogeneous studies, involving only 346 patients in total, and might not be large enough to draw strong, generalizable conclusions. This limits the comprehensiveness of findings and may not provide sufficient power for robust conclusions. In addition, the heterogeneity in study designs and measurement techniques considerably undermines the comparability and reliability of the findings. Specifically, pulmonary pressures were assessed using different methodologies: one study utilized continuous monitoring via a CardioMEMS device, two employed RHC, and three relied on echocardiography. While CardioMEMS and RHC are regarded as more accurate and reliable, echocardiography is inherently operator-dependent and may be subject to variability in measurement accuracy. Furthermore, several studies derived mPAP using estimation equations rather than direct measurement, thereby introducing additional uncertainty into the results. This variability in both measurement techniques and study designs—including differences in follow-up duration, patient selection criteria (e.g., HFpEF vs. HFrEF), and endpoints (rest vs. exercise hemodynamics)—complicates direct comparisons across studies and limits the generalizability of our conclusions. These limitations affect the strength of the conclusions and suggest the need for more uniform methodologies and larger trials to further clarify the effects of SGLT2 inhibitors on PAPs.

Despite these limitations, the study also has several strengths. The use of a comprehensive and systematic review process, following PRISMA guidelines, ensured a rigorous and transparent selection of relevant studies. Additionally, the inclusion of studies with multiple measurement techniques provides a broad perspective on the impact of SGLT2 inhibitors across different settings and patient populations. Furthermore, the study's focus on exploring the effects of SGLT2 inhibitors on pulmonary hemodynamics in HF, an area with limited prior research, addresses an important clinical gap and offers valuable insights for future studies.

To enhance the robustness and clinical applicability of future research, it is imperative to adopt standardized methodologies. We recommend that future studies employ direct measurement methods such as RHC or validated implantable devices like CardioMEMS rather than relying solely on echocardiographic estimations or derived calculations. Larger, multicenter trials are encouraged as these will improve statistical power and provide more definitive insights into the impact of SGLT2 inhibitors on pulmonary hemodynamics. Consistent recording and calculation of key parameters, such as mPAP, PVR, and PCWP, are essential to enhance the depth and comparability of results. Longer follow-up periods are necessary to evaluate the sustained impact of SGLT2 inhibitors on pulmonary pressures and related outcomes. By addressing these methodological concerns, future research can yield more reliable and generalizable findings, ultimately advancing our understanding and management of PH in HF patients.

## 5. Conclusion

In conclusion, this systematic review highlights the potential benefits of SGLT2 inhibitors, particularly DAPA and EMPA, in improving pulmonary hemodynamics and reducing PAPs in HF patients. The review demonstrated that these drugs show promising results in reducing mPAP and PASP. This effect highlights their potential to improve outcomes for patients with HF, particularly those with elevated pulmonary pressures.

Additionally, reductions in key markers such as PCWP and NT-proBNP further suggest that SGLT2 inhibitors can play a significant role in managing PH and HF. However, the difference in the outcome for trials using DAPA and those with EMPA, as well as between rest and exercise conditions, suggests these drugs may have distinct effects on pulmonary hemodynamics, and these effects may vary based on the physiological setting warranting further research. While both drugs show promise, the current evidence base comprising a handful of studies for each drug may not be sufficient to draw definitive conclusions. It is important to acknowledge that not all research has employed the gold standard methods for measuring pulmonary pressures. More comparative research between DAPA and EMPA, especially in diverse clinical settings, is needed to fully understand their differential effects on rest versus exercise hemodynamics. Overall, this review reinforces the growing evidence supporting the use of SGLT2 inhibitors in managing pulmonary pressures and improving cardiovascular outcomes in HF patients.

## Figures and Tables

**Table 1:** 

	**Inclusion criteria**	**Exclusion criteria**
Participants/population	Heart failure patients (preserved/reduced EF) on SGLT2 inhibitors.	Non-heart failure populations; HF patients without SGLT2 inhibitor use.
Intervention/exposure	1. The primary intervention must be the administration of SGLT2 inhibitors. 2. Examine the effect of an SGLT2 inhibitor on the mean pulmonary artery pressure (mPAP) or Pulmonary artery systolic pressure (PASP).	1. Studies where SGLT2 inhibitors are not the primary intervention or are used in combination with other novel or investigational drugs that might confound results. 2. Lack of sufficient data to establish a meaningful relationship between the change in mPAP or PASP with the administration of SGLT2 inhibitor drug in heart failure patients. 3. Studies focusing solely on other pulmonary artery hemodynamic variables such as pulmonary artery diastolic pressure (PADP), pulmonary capillary wedge pressure (PCWP), pulmonary vascular resistance (PVR), or N-terminal pro-B-type Natriuretic Peptide (NT-proBNP).
Comparator/control	Placebo or standard care without SGLT2 inhibitors.	Studies lacking a comparator group or not directly comparing SGLT2 inhibitors with placebo/standard care.
Types of study	RCTs, observational studies, cohort studies, human studies in adults (≥ 18 years), and English.	Review articles, opinion pieces, case reports, animal studies, non-English articles, insufficient data, and studies with very short follow-up (< 1 week).

**Table 2:** Baseline characteristics of the included studies.

**SN**	**Author**	**Year**	**Study design**	**Drug used**	**HF type**	**Sample size**	**Follow-up duration**	**Mean age**	**Baseline medications**	**Gender**	**Comorbidities**	**Mean left ventricular ejection fraction (LVEF) (** **m** **e** **a** **n** ± %**)**	**Mean NT-proBNP level median (IQR) (pg/mL)**	**NYHA functional class**
1	Reis et al. [12]	2022	RCT	Dapagliflozin 10 mg vs. placebo	HFrEF	40 (DAPA group 20 vs. control group 20)	24 weeks	DAPA group: 60.3 ± 11.6; control: 61.7 ± 14.8	Optimum medical therapy for heart failure	DAPA group: Male 85% vs. female 15%; control: Male: 80% vs. 20% female	Hypertension 25 (62.5%), dyslipidemia 26 (65.0%),, atrial fibrillation 12 (30.0%), chronic kidney disease 8 (20.0%), peripheral artery disease 9 (22.5%), and COPD 11 (27.5%)	34.1 ± 8.3%	781.0 (350.7–1599.1) pg/mL	Classes III–IV = 8 (20%)
2	Reddy et al. [13]	2024	RCT	Dapagliflozin 10 mg	HFpEF	38 (DAPA group 21 vs. placebo 17)	24 weeks	67.4 ± 8.5 in both DAPA and control groups	Optimum medical therapy for heart failure	DAPA group: 33% male vs. 67% female; control group: 35% male vs. 65% female	Obesity 27 (71%) and atrial fibrillation 13 (35.14%)	Placebo group = 63%; dapagliflozin group = 61%	Placebo group = 118 (76–226); dapagliflozin group = 235 (102–394)	[Placebo: II = 5 (29%) vs. III = 12 (71%)]; [DAPA group: II = 7 (33%) vs. III = 14 (67%)]
3	Omar et al. [14]	2020	RCT	Empagliflozin 10 mg	HFrEF	70	12 weeks	Empagliflozin = 59 ± 8; placebo = 56 ± 11	Optimum medical therapy for heart failure and 12 participants were on glucose lowering medications	Empagliflozin = male 32 (91%); placebo = male 31 (89%)	Empagliflozin = [diabetes mellitus Type 2 = 5 (14%), undiagnosed type 2 diabetes = 5 (7%), atrial fibrillation = 10 (29%), ischemic heart disease = 14 (40%), chronic kidney disease = 1 (3%)]; Placebo = [diabetes mellitus Type 2 = 7 (20%), undiagnosed Type 2 diabetes = 1 (1%), atrial fibrillation = 8 (23%), ischemic heart disease = 14 (40%), chronic kidney disease = 3 (9%)]	Empagliflozin = 25 ± 8%; placebo = 28 ± 7%	Empagliflozin = 428 (246–944) ng/L; placebo = 510 (209–966) ng/L	Empagliflozin = [NYHA II 27 (77%), NYHA III 8 (23%)]; placebo = [NYHA II 31 (89%), NYHA III 4 (11%)]
4	Nassif et al. [15]	2021	RCT	Empagliflozin 10 mg	HF (irrelevant of EF)	65 (33 EMPA and 32 placebo)	12 weeks	Empagliflozin = 69.5 ± 12.0; placebo = 62.9 ± 13.3	Optimum medical therapy for heart failure	Empagliflozin = male 21 (63.6%); placebo = male 20 (62.5%)	Empagliflozin = ischemic heart disease 13 (39.4%), T2DM 18 (54.5%), atrial fibrillation 20 (60.6%); placebo = ischemic heart disease 10 (31.3%), T2D 16 (50.0%), atrial fibrillation 17 (53.1%)	Empagliflozin = 46.7 ± 14.9%; placebo = 40.7 ± 17.2%	Empagliflozin = 865.5 (311.0, 1982.5); placebo = 563.5 (153.0, 1964.0)	Empagliflozin = [NYHA Class II 14 (42.4%), NYHA Class III 18 (54.5%)]; placebo = [NYHA Class II 16 (50.0%), NYHA Class III 16 (50.0%)]
5	Jariwala and Gururaj [16]	2023	Retrospective observational study	Empagliflozin + OMT vs. OMT	Right heart failure (irrelevant to EF)	55	36 weeks	N/A	Optimum medical therapy for heart failure	N/A	COPD 63.4%, ILD 23.5%, pulmonary fibrosis 2.3%, hypersensitivity pneumonitis 2.6%, unilateral pneumonectomy/lung atresia 0.4%, miscellaneous lung diseases 7.2%	N/A	2345 ± 405.67 pg/dL	N/A
6	Correale et al. [17]	2023	Prospective cohort	SGLT2i	Heart failure (irrelevant to EF)	78 (38 SGLT2i vs. 40 previous antidiabetic)	12 weeks	65 ± 7 in SGLT2i group vs. 70 ± 9 in OMT group	Optimum medical therapy for heart failure and glucose lowering medications	SGLT2i group: Male 89% vs. female 11%; control group: Male 0% vs. female 20%	T2DM 100%, COPD 22%, atrial fibrillation 40%, hypertension 75%, anemia 6%	Mean LVEF for SGLT2 inhibitors: 45 ± 9% Mean LVEF for controls: 38 ± 8% Statistical significance: *p* < 0.001	NT-proBNP for SGLT2 inhibitors: 1422 ± 2181 pg/mL NT-proBNP for other antidiabetic drugs: 1264 ± 3172 pg/mL Statistical significance: Not significant (ns)	NYHA Class II = 62%; NYHA Class III = 33%

**Table 3:** The method of pulmonary artery pressure assessment in the included studies.

**Pulmonary artery pressure measurement methods**	**Studies**
Echocardiography	• Reis et al. [12] • Jariwala and Gururaj [16] • Correale et al. [17]
Right heart catheterization	• Reddy et al. [13] • Omar et al. [14]
Pulmonary artery pressure sensor (CardioMEMS)	• Nassif et al. [15]

**Table 4:** Primary outcome from the included studies demonstrating changes in mPAP and PASP values in intervention versus control group. SGLT2i, sodium–glucose cotransporter 2 inhibitors; mPAP, mean pulmonary artery pressure; PASP, pulmonary artery systolic pressure; OMT, optimum medical therapy; HFrEF, heart failure with reduced ejection fraction; HFpEF, heart failure with preserved ejection fraction; N/R, not recordable.

**SN**	**Author**	**Year**	**Trial**	**HF type**	**Total number of participants**	**Duration of follow-up**	**Results**	
1	Reis et al. [12]	2022	Dapagliflozin 10 mg vs. placebo	HFrEF	40	6 months	PASP	DAPA group: Baseline PASP = 36.6 ± 7.8 mmHg Follow-up PASP = 30.7 ± 8.6 mmHg Mean change: −5.9 mmHg Control group: Baseline PASP = 29.8 ± 11.4 mmHg Follow-up PASP = 39.4 ± 10.4 mmHg Mean change: +9.6 mmHg *p* value interaction between drug and placebo (*p* < 0.001)
							MPAP Derived mPAP = 0.61 × PASP+1.95	DAPA Group: Baseline mPAP = 24.27 ± 4.76 mmHg, Follow-up mPAP = 20.33 ± 5.24 mmHg Mean change: -3.94 mmHg CONTROL group, Baseline mPAP = 20.13 ± 6.95 mmHg, Follow-up mPAP = 25.99 ± 6.34 Mean change: +5.86 mmHg

2	Reddy et al. [13]	2024	Dapagliflozin 10 mg	HFpEF	37	24 weeks	MPAP	Rest hemodynamics: (mean change from baseline) DAPA group (baseline vs. follow-up): Mean change: −1.8 mmHg (SD = 4.0 mmHg) Control group (baseline vs. follow-up): Mean change: 1.1 mmHg (SD = 7.4 mmHg Intervention vs. control: Mean change: −2.9 mmHg (−6.9 to 1.1 mmHg), *p* = 0.13 Exercise hemodynamics: DAPA group (baseline vs. follow-up): Mean change: −5.2 mmHg (SD = 7.0 mmHg) Control group (baseline vs. follow-up): Mean change: 0.7 mmHg (SD = 8.7 mmHg) Intervention vs. control: Mean change: −5.9 mmHg (−11.0 to −0.69 mmHg), *p* = 0.02
							PASP	Rest hemodynamics: (mean change from baseline) DAPA group: Mean change: −2.2 mmHg (SD = 5.4 mmHg) Control group: Mean change: 0.3 mmHg (SD = 8.5 mmHg) Intervention vs. control: Mean change: −2.5 mmHg (−7.2 to 2.3 mmHg), *p* = 0.27 Exercise hemodynamics: DAPA group: Mean change: −5.4 mmHg (SD = 8 mmHg) Control group: Mean change: −0.1 mmHg (SD = 10.3 mmHg) Intervention vs. control: Mean change: −5.3 mmHg (−11.4 to 0.8 mmHg), *p* = 0.08

3	Omar et al. [14]	2020	Empagliflozin 10 mg	HFrEF	70	12 weeks	MPAP	Rest subgroup: (mean change from baseline) EMPA group: Mean change: −1.1 9 mmHg (95% CI: −3.13 to 0.75, *p* = 0.23) Control group: Mean change: −1.38 mmHg (95% CI: −3.32 to 0.56, *p* = 0.16) Intervention vs. control: Mean change: 0.19 mmHg (95% CI: −2.37 to 2.75, *p* = 0.89) Exercise hemodynamics: EMPA group: Mean change: −2.37 mmHg (95% CI: −4.80 to 0.06, *p* = 0.056) Control group: Mean change: −1.13 mmHg (95% CI: −3.55 to 1.30, *p* = 0.36) Intervention vs. control: Mean change: −1.24 mmHg (95% CI: −4.55 to 2.07, *p* = 0.48)

4	Nassif et al. [15]	2021	Empagliflozin 10 mg	HF (irrelevant of EF)	65 (33 EMPA 32 placebo)	12 weeks	MPAP	Mean change from baseline Intervention vs. control: −1.8 mmHg (95% CI: −3.30 to −0.30 mmHg), *p* value of 0.21.
							PASP	Mean change from baseline Intervention vs. control: −2.0 mmHg (95% CI: −4.8 to 0.8 mmHg), *p* value of 0.16.

5	Jariwala and Gururaj [16]	2023	Empagliflozin + optimum medical therapy (OMT) vs. OMT	Right heart failure (irrelevant of EF)	55	9 months	PASP	The measurement of PASP exhibited minimal variation (−2.392 mm, *p* = 0.723) and remained rather constant.

6	Correale et al. [17]	2023	SGLT2i	Heart Failure (irrelevant of EF)	78 (38 SGLT2i vs. 40 previous antidiabetic)	3 months	PASP	SGLT2i group: Baseline PASP: 30.6 ± 8.8 mmHg Follow-up PASP: 24 ± 8.4 mmHg Mean change: −6.6 mmHg, *p* < 0.001 Control: Baseline PASP: 30.3 ± 9.5 mmHg Follow-up PASP: 30.2 ± 10.7 mmHg Mean change: −0.1, *p* = not statistically significant. Intervention vs. control: −6.5 (−12.38 to −0.62), *p* = 0.03
							MPAP derived	In the SGLT2i group, the baseline mPAP (derived) was 21.86 ± 4.9 mmHg, which decreased to 16.59 ± 5.5 mmHg after 3 months. In contrast, the non-SGLT2i group baseline mPAP (derived) was 20.65 ± 5.5 mmHg and mPAP (derived) after 3 months was calculated to be 20.65 ± 6.71 mmHg

**Table 5:** Subgroup analysis demonstrating changes in mPAP and PASP values in rest versus exercise. SGLT2i, sodium–glucose cotransporter 2 inhibitors; mPAP, mean pulmonary artery pressure; PASP, pulmonary artery systolic pressure; HFrEF, heart failure with reduced ejection fraction; HFpEF, heart failure with preserved ejection fraction; N/R, not recordable.

**Studies**	**Sample size**	**Follow-up duration**	**MPAP**		**PASP**	
			**Rest**	**Exercise**	**Rest**	**Exercise**
Reddy et al. [13]	37	24 weeks	DAPA group (baseline vs. follow-up): Mean change: −1.8 mmHg (SD = 4.0 mmHg) Control group (baseline vs. follow-up): Mean change: 1.1 mmHg (SD = 7.4 mmHg Intervention vs. control: Mean change: −2.9 mmHg (−6.9 to 1.1 mmHg), *p* = 0.13	DAPA group (baseline vs. follow-up): Mean change: −5.2 mmHg (SD = 7.0 mmHg) Control group (baseline vs. follow-up): Mean change: 0.7 mmHg (SD = 8.7 mmHg) Intervention vs. control: Mean change: −5.9 mmHg (−11.0 to−0.69 mmHg), *p* = 0.02	(Mean change from baseline) DAPA group: Mean change: −2.2 mmHg (SD = 5.4 mmHg) Control group: Mean change: 0.3 mmHg (SD = 8.5 mmHg) Intervention vs. control: Mean change: −2.5 mmHg (−7.2 to 2.3 mmHg), *p* = 0.27	(Mean change from baseline) DAPA group: Mean change: −5.4 mmHg (SD = 8 mmHg) Control group: Mean change: −0.1 mmHg (SD = 10.3 mmHg) Intervention vs. control: Mean change: −5.3 mmHg (−11.4 to 0.8 mmHg), *p* = 0.08

Omar et al. [14]	70	12 weeks	EMPA group: Mean change: −1.1 9 mmHg (95% CI: −3.13 to 0.75, *p* = 0.23) Control group: Mean change: −1.38 mmHg (95% CI: −3.32 to 0.56, *p* = 0.16) Intervention vs. control: Mean change: 0.19 mmHg (95% CI: −2.37 to 2.75, *p* = 0.89)	EMPA group: Mean change: −2.37 mmHg (95% CI: −4.80 to 0.06, *p* = 0.056) Control group: Mean change: −1.13 mmHg (95% CI: −3.55 to 1.30, *p* = 0.36) Intervention vs. control: Mean change: −1.24 mmHg (95% CI: −4.55 to 2.07, *p* = 0.48)	N/R	N/R

**Table 6:** Subgroup analysis demonstrating changes in mPAP and PASP values in dapagliflozin versus empagliflozin. SGLT2i, sodium–glucose cotransporter 2 inhibitors; mPAP, mean pulmonary artery pressure; PASP, pulmonary artery systolic pressure; HFrEF, heart failure with reduced ejection fraction; HFpEF, heart failure with preserved ejection fraction; N/R, not recordable.

**Dapagliflozin**		**Empagliflozin**	
**Study**	**Parameters**	**Study**	**Parameters**
Reis et al. [12]	PASP DAPA group: Baseline PASP = 36.6 ± 7.8 mmHg Follow-up PASP = 30.7 ± 8.6 mmHg Mean change: −5.9 mmHg. Control group: Baseline PASP = 29.8 ± 11.4 mmHg Follow-up PASP = 39.4 ± 10.4 mmHg Mean change: +9.6 mmHg *p* value interaction between drug and placebo (*p* < 0.001)	Omar et al. [14]	MPAP Rest subgroup: (mean change from baseline) EMPA group: Mean change: −1.1 9 mmHg (95% CI: −3.13 to 0.75, *p* = 0.23) Control group: Mean change: −1.38 mmHg (95% CI: −3.32 to 0.56, *p* = 0.16) Intervention vs. control: Mean change: 0.19 mmHg (95% CI: −2.37 to 2.75, *p* = 0.89) Exercise hemodynamics: EMPA group: Mean change: −2.37 mmHg (95% CI: −4.80 to 0.06, *p* = 0.056) Control group: Mean change: −1.13 mmHg (95% CI: −3.55 to 1.30, *p* = 0.36) Intervention vs. control: Mean change: −1.24 mmHg (95% CI: −4.55 to 2.07, *p* = 0.48)

Reddy et al. [13]	PASP Rest hemodynamics: (Mean change from baseline) DAPA group: Mean change: −2.2 mmHg (SD = 5.4 mmHg) Control group: Mean change: 0.3 mmHg (SD = 8.5 mmHg) Intervention vs. control: Mean change: −2.5 mmHg (−7.2 to 2.3 mmHg), *p* = 0.27 Exercise hemodynamics: DAPA group: Mean change: −5.4 mmHg (SD = 8 mmHg) Control group: Mean change: −0.1 mmHg (SD = 10.3 mmHg) Intervention vs. control: Mean change: −5.3 mmHg (−11.4 to 0.8 mmHg), *p* = 0.08 mPAP Rest hemodynamics: DAPA group (baseline vs. follow-up): Mean change: −1.8 mmHg (SD = 4.0 mmHg) Control group (baseline vs. follow-up): Mean change: 1.1 mmHg (SD = 7.4 mmHg Intervention vs. control: Mean change: −2.9 mmHg (−6.9 to 1.1 mmHg), *p* = 0.13 Exercise hemodynamics: DAPA group (baseline vs. follow-up): Mean change: −5.2 mmHg (SD = 7.0 mmHg) Control group (baseline vs. follow-up): Mean change: 0.7 mmHg (SD = 8.7 mmHg) Intervention vs. control: Mean change: −5.9 mmHg (−11.0 to −0.69 mmHg), *p* = 0.02	Nassif et al. [15]	PASP Mean change from baseline Intervention vs. control: −1.8 mmHg (95% CI: −3.30 to −0.30 mmHg), *p* value of 0.21 mPAP Mean change from baseline Intervention vs. control: −1.9 mmHg (95% CI: −4.5 to 0.8 mmHg), *p* value of 0.21

		Jariwala and Gururaj [16]	PASP The measurement of PASP exhibited minimal variation (−2.392 mm, *p* = 0.723) and remained rather constant.

**Table 7:** Subgroup analysis demonstrating changes in mPAP and PASP values in HFpEF versus HFrEF groups. SGLT2i, sodium–glucose cotransporter 2 inhibitors; mPAP, mean pulmonary artery pressure; PASP, pulmonary artery systolic pressure; HFrEF, heart failure with reduced ejection fraction; HFpEF, heart failure with preserved ejection fraction; N/R, not recordable.

**HFrEF**		**HFpEF**	
**Study**	**Parameters**	**Study**	**Parameters**
Reis et al. [12]	PASP DAPA group: Baseline PASP = 36.6 ± 7.8 mmHg Follow-up PASP = 30.7 ± 8.6 mmHg Mean change: −5.9 mmHg Control group: Baseline PASP = 29.8 ± 11.4 mmHg Follow-up PASP = 39.4 ± 10.4 mmHg Mean change: +9.6 mmHg *p* value interaction between drug and placebo (*p* < 0.001)	Reddy et al. [13]	PASP Rest hemodynamics: (Mean change from baseline) DAPA group: Mean change: −2.2 mmHg (SD = 5.4 mmHg) Control group: Mean change: 0.3 mmHg (SD = 8.5 mmHg) Intervention vs. control: Mean change: −2.5 mmHg (−7.2 to 2.3 mmHg), *p* = 0.27 Exercise hemodynamics: DAPA group: Mean change: −5.4 mmHg (SD = 8 mmHg) Control group: Mean change: −0.1 mmHg (SD = 10.3 mmHg) Intervention vs. control: Mean change: −5.3 mmHg (−11.4 to 0.8 mmHg), *p* = 0.08 mPAP Rest hemodynamics: DAPA group (baseline vs. follow-up): Mean change: −1.8 mmHg (SD = 4.0 mmHg) Control group (baseline vs. follow-up): Mean change: 1.1 mmHg (SD = 7.4 mmHg Intervention vs. control: Mean change: −2.9 mmHg (−6.9 to 1.1 mmHg), *p* = 0.13 Exercise hemodynamics: DAPA group (baseline vs. follow-up): Mean change: −5.2 mmHg (SD = 7.0 mmHg) Control group (baseline vs. follow-up): Mean change: 0.7 mmHg (SD = 8.7 mmHg) Intervention vs. control: Mean change: −5.9 mmHg (−11.0 to−0.69 mmHg), *p* = 0.02

Omar et al. [14]	MPAP Rest subgroup: (mean change from baseline) EMPA group: Mean change: −1.1 9 mmHg (95% CI: −3.13 to 0.75, *p* = 0.23) Control group: Mean change: −1.38 mmHg (95% CI: −3.32 to 0.56, *p* = 0.16) Intervention vs. control: Mean change: 0.19 mmHg (95% CI: −2.37 to 2.75, *p* = 0.89) Exercise hemodynamics: EMPA group: Mean change: −2.37 mmHg (95% CI: −4.80 to 0.06, *p* = 0.056) Control group: Mean change: −1.13 mmHg (95% CI: −3.55 to 1.30, *p* = 0.36) Intervention vs. control: Mean change: −1.24 mmHg (95% CI: −4.55 to 2.07, *p* = 0.48)		

**Table 8:** Summary of secondary outcomes for SGLT2 inhibitors in improving pulmonary hemodynamics (PADP, PVR, PCWP, and NT-proBNP) extracted from our included studies. SGLT2i, sodium–glucose cotransporter 2 inhibitors; PADP, pulmonary artery diastolic pressure; PCWP, pulmonary capillary wedge pressure; NT-proBNP, N-terminal pro-B-type natriuretic peptide; PVR, pulmonary vascular resistance; CI, confidence interval; RVs'/PA Ea, RVs' to pulmonary arterial effective elastance (Ea); TAPSE, tricuspid annular plane systolic excursion; PASP, pulmonary artery systolic pressure.

**SN**	**Author**	**PADP**	**PCWP**	**NT-proBNP**	**PVR**	**Right ventricle pulmonary artery coupling**
1	Reis et al. [12]			A greater reduction in NT-proBNP levels with dapagliflozin vs. control after 6 months (−217.6 vs. 650.3 pg/mL, *p* = 0.007)		Dapagliflozin group: TAPSE increased (19.0→20.2 mm): Better RV systolic function. PASP decreased (36.6→30.7 mmHg): Reduced pulmonary pressure. TAPSE/PASP increased by an estimate (0.52→0.66): Improved RV–PA coupling. Control group: TAPSE stayed almost the same (18.6→18.7 mm): No RV improvement. PASP increased (29.8→39.4 mmHg): Worse pulmonary pressure. TAPSE/PASP decreased (0.62→0.47): Worsened RV–PA coupling. Indicates deterioration in RV's ability to handle pressure load.

2	Reddy et al. [13]		Rest hemodynamics: The change in PCWP after 24 weeks was −3.5 mmHg (95% CI: −6.8 to −0.3, *p* = 0.03) for dapagliflozin compared to placebo, with placebo showing a change of +1.1 mmHg and dapagliflozin showing a change of −2.5 mmHg, which was statistically significant. Exercise hemodynamics: Dapagliflozin resulted in a statistically significant reduction in PCWP after 24 weeks, with a change of −6.1 mmHg (95% CI: −11.3 to −0.9, *p* = 0.02) compared to placebo. The placebo group showed a change of −0.4 mmHg at 24 weeks, while the dapagliflozin group demonstrated a significant reduction of −6.6 mmHg at 24 weeks		Rest hemodynamics: The change in PVR after 24 weeks for dapagliflozin compared to placebo was 0.1 mmHg (95% CI: −0.3 to 0.6, *p* = 0.58), with placebo showing a change of +0.3 mmHg and dapagliflozin showing a change of 0.3 mmHg from baseline to follow-up, which was not statistically significant. Exercise hemodynamics: The change in PVR for dapagliflozin compared to placebo after 24 weeks was 0.1 mmHg (95% CI: −0.3 to 0.6, *p* = 0.58), with placebo showing a change of +0.2 mmHg and dapagliflozin showing a change of 0.3 mmHg from baseline to follow-up, and this difference was also not statistically significant	RVs'/PA Ea, cm·s^−1^/mmHg·mL^−1^ Rest hemodynamics: A reduction of RVs'/PA Ea (SD) in treatment vs. control −1.5 (7.4) vs. −2.6 (10.1) with difference of −1.1 (−7.5 to 5.4) between the groups (*p* = 0.74) Exercise hemodynamics: A greater reduction of RVs'/PA Ea (SD) in treatment vs. control −4.0 (4.5) vs. 2.4 (8.1) with difference of 6.3 (0.2–12.5) between the groups (*p* = 0.04) Conclusion: At rest, both groups showed slight worsening (reduction) in RV–PA coupling. and the difference is not statistically significant (*p* = 0.74). No measurable benefit of treatment over control at rest in terms of RV–PA coupling. During exercise, the treatment group showed a greater reduction in RVs'/PA Ea (statistically significant), whereas the control group's coupling worsened, which reflects better physiological adaptation with treatment

3	Omar et al. [14]	Resting hemodynamics: EMPA group: PADP baseline: 14 ± 5 mmHg and follow-up: 13 ± 6 mmHg **Control group:** PADP baseline: 12 ± 5 mmHg vs. follow-up: 11 ± 5 mmHg. Exercise hemodynamics: EMPA group: PADP baseline: 32 ± 9 mmHg and follow-up: 29 ± 7 mmHg Control group: PADP baseline: 26 ± 7 mmHg vs. follow-up 25 ± 9 mmHg	REST: EMPA group: PCWP baseline vs. follow-up (−2.16 [−3.84 to −0.47] mmHg; *p*: 0.012) vs. control group baseline vs. follow-up (−0.69 [−2.38 to 1.00] mmHg; *p*: 0.424). Mean change of PCWP between the EMPA group and placebo at follow-up (−1.47 [−3.86 to 0.82] mmHg; *p*: 0.228) Exercise: EMPA group: PCWP baseline vs. follow-up (−4.14 [−6.86 to −1.42] mmHg; *p*: 0.003) vs. control group PCWP baseline vs. follow-up (−0.64 [−3.32 to 2.04] mmHg; *p*: 0.640). Mean change of PCWP between EMPA group and placebo at follow-up (−3.50 [−7.32 to 0.32] mmHg; *p*: 0.073)			N/A

4	Nassif et al. [15]	At Veek 12, PA diastolic pressure was 1.7 mmHg lower (95% CI, 0.3–3.2; *p* = 0.02) in empagliflozin group compared to placebo group		A significantly greater proportion of patients treated with empagliflozin vs. placebo experienced ≥ 20% reduction in NT-proBNP at 12 weeks (34% EMPA vs. 7% placebo group; adjusted odds ratio, 15.4 [95% CI, 1.7–131.6]; *p* = 0.01)		N/A

5	Jariwala and Gururaj [16]			There was significant improvement in NT-proBNP levels in empagliflozin group (baseline to follow-up) (2345 ± 405.67 to 789.45 ± 233.89 pg/dL, *p* < 0.001)		RV–PC coupling (TAPSE/PASP) (mean, SD) mm/mmHg in EMPA + OMT group was 0.252 ± 0.080 mm/mmHg at baseline and posttreatment it improved to 0.300 ± 0.070 mm/mmHg. The TAPSE increase (*p* = 0.076) suggests a trend toward significance, while PASP remained unchanged (*p* = 0.723), so the change in coupling is mostly driven by RV functional improvement. TAPSE **↑** and PASP ≈ constant in OMT + empagliflozin group. OMT group showed less or no significant change in TAPSE or PASP

6	Correale et al. [17]					Gliflozin group: TAPSE ↑ (19.4→21.2): Improved RV systolic function. PASP ↓ (30.6→24.0): Lower pulmonary afterload. TAPSE/PASP ↑ (0.63→0.88). Marked improvement in RV–PA coupling, reflecting a more efficient right heart adaptation to a reduced pulmonary pressure. Control group: TAPSE ≈ stable (18.2→18.4): No meaningful RV functional change. PASP ≈ stable (30.3→30.2): No change in pulmonary pressure. TAPSE/PASP (0.60→0.61): No meaningful improvement in coupling

**Table 9:** Summary of clinical evidence for SGLT2 inhibitors in improving pulmonary hemodynamics in several observational studies. SGLT2i, sodium–glucose cotransporter 2 inhibitors; mPAP, mean pulmonary artery pressure; PASP, pulmonary artery systolic pressure; HFrEF, heart failure with reduced ejection fraction; HFpEF, heart failure with preserved ejection fraction.

**Study**	**Study type**	**Main findings**
Palmiero et al. [18]	Multicenter, prospective observational cohort study involving 31 patients with Type 2 diabetes mellitus and chronic heart failure with reduced ejection fraction (HFrEF; LVEF ≤ 40%) enrolled between February 2021 and June 2022. Patients were started on empagliflozin 10 mg once daily after an adequate period of optimal medical therapy (to avoid excessive fluctuations of blood pressure and volemic state). Follow-up period: 6 months	• PASP at baseline vs. 6 months after empagliflozin mean (SD): 35.23 (14.61) vs. 28.52 (6.91) *p* = 0.024. • NT-proBNP, median [IQR] at baseline 1159.00 [892.50, 2758.50] vs. 6 months 550.00 [330.50, 1610.00], *p* = 0.040

Mohanakrishnan et al. [19]	A retrospective, crossover study design on 16 congestive heart failure patients with implanted pulmonary artery pressure sensors who have been recently started on SGLT2 inhibitors. The goal was to compare the changes in pulmonary artery diastolic pressures pre and post SGLT2 inhibitors usage. Follow-up period: 4 weeks	• SGLT2 inhibitors significantly reduced PADP by 1.71 mmHg lower (95% CI, *p* = 0.003) when comparing pre- vs. postpulmonary artery pressures. • The overall mean PADP was 6.47% lower than the baseline pressure

Kirschbaum et al. [20]	Single center, nonrandomized retrospective observational study of 17 ambulatory HFrEF or HFpEF patients with a CardioMEMS device, in whom either dapagliflozin or empagliflozin was initiated at the discretion of the treating physician. PAP readings were collected daily starting 4 weeks prior to SGLT2i initiation for up to 10 weeks following initiation of treatment with SGLT2i	• Statistically significant reduction in PASP (−3.59 ± 1.55 mmHg; *p* = 0.034), mPAP (−3.06 ± 1.22 mmHg; *p* = 0.014), and PADP (−2.65 ± 0.98 mmHg; *p* = 0.008)

Hassan et al. [21]	A single-center, prospective study started in October 2021 including 23 patients with nonischemic dilated cardiomyopathy (NIDCM). The study evaluated the effect of dapagliflozin 10 mg on pulmonary artery pressure via right heart catheterization after a follow-up period of 6 months	• A statistically significant decrease in PASP in baseline vs. follow-up (median +/IQ range) (44 [32–58] vs. 40 [31–45] mmHg *p* = 0.035), mPAP (31.3 [±10.5] vs. 27.7 [±7.3] mmHg, *p* = 0.03), and PADP (25 [17–34] vs. 20 [17–23] mmHg, *p* = 0.005) • A mild decrease in PVR (baseline vs. follow-up) Wood units (WU) 2.7 (±1.59) vs. 2.1 (±1.59), *p* = 0.596

Çamcı and Yılmaz [22]	Retrospective study including 168 HFrEF patients with New York Heart Association (NYHA) class ≥ 2 symptoms despite optimal medical treatment and who were recently started on SGLT2 inhibitor therapy. Follow-up period: 6 months	• A significant improvement in mean pulmonary artery pressure (mPAP) (baseline vs. follow-up) (39.6 ± 7.8 mmHg vs. 32 ± 6.8 mmHg, *p* = 0.003). • A significant drop in NT-proBNP levels (picograms per milliliter) (baseline vs. follow-up) (2876 ± 401 vs. 1034 ± 361, *p* < 0.001)

Mullens et al. [23]	Single-center open label observational trial that investigated the short-term effects of dapagliflozin in nine HFrEF patients under optimum guideline-directed therapy with elevated PAP between October and December 2019, previously implanted with CardioMEMS or Cordella Sensor. Follow-up period: 7 days before and after starting dapagliflozin relative to the first day of each period	• The mPAP was reduced from 42 ± 9.16 to 38 ± 9.95 mmHg with dapagliflozin therapy (*p* < 0.05) • The drop in PAP occurred within the first 2 days of dapagliflozin and remained stable for the week following the start of the therapy

Battistoni et al. [24]	Retrospective multicenter study involving 95 HFrEF patients who were initiated on dapagliflozin therapy in an outpatient setting were followed-up for 6 months	• PASP reduced from 32 ± 10 vs. 28 ± 7 (*p* < 0.001)

## Data Availability

The data supporting the findings of the manuscript will be made available upon request to the corresponding author.

## Nomenclature

HF: heart failure
EF: ejection fraction
HFrEF: heart failure with reduced ejection fraction
HFpEF: heart failure with preserved ejection fraction
PAPs: pulmonary artery pressures
PH: pulmonary hypertension
RV: right ventricle
mPAP: mean pulmonary artery pressure
SGLT2is: sodium–glucose cotransporter 2 inhibitors
PASP: pulmonary artery systolic pressure
RCTs: randomized control trials
NT-proBNP: N-terminal pro-B-type natriuretic peptide
PCWP: pulmonary capillary wedge pressure
PADP: pulmonary artery diastolic pressure
PVR: pulmonary vascular resistance
NYHA: New York Heart Association
DAPA: dapagliflozin
EMPA: empagliflozin
RHC: right heart catheterization
ROB 2: Cochrane Risk of Bias 2
NOS: Newcastle–Ottawa Scale
OMT: optimum medical therapy
KCCQ-OS: Kansas City Cardiomyopathy Questionnaire Overall Summary
PH-LHD: pulmonary hypertension secondary to left heart disease
LVEF: left ventricle ejection fraction
NIDCM: nonischemic dilated cardiomyopathy
RVSP: right ventricular systolic pressure
PAH: pulmonary arterial hypertension
PA: pulmonary artery
IpcPH: isolated postcapillary pulmonary hypertension
CpcPH: combined post- and precapillary PH
RV–PA coupling: right ventricle pulmonary artery coupling
CPETs: cardiopulmonary exercise tests
MACE: major adverse cardiac events
ICAM-1: intercellular adhesion molecule-1
MCP-1: monocyte chemoattractant protein-1

## Supporting Information

Supporting Information Additional supporting information can be found online in the Supporting Information section. Appendix S1: PRISMA checklist. Appendix S2: Search strategy. Appendix S3: Quality Assessment—Cochrane Risk of Bias 2 (Appendix 3.1) and Newcastle–Ottawa Scale (Appendix 3.2). Appendix S4: AMSTAR checklist.
